# Depth of Encoding Through Observed Gestures in Foreign Language Word Learning

**DOI:** 10.3389/fpsyg.2019.00033

**Published:** 2019-01-29

**Authors:** Manuela Macedonia, Claudia Repetto, Anja Ischebeck, Karsten Mueller

**Affiliations:** ^1^Department of Information Engineering, Johannes Kepler University Linz, Linz, Austria; ^2^Research Group Neural Mechanisms of Human Communication, Max Planck Institute for Human Cognitive and Brain Sciences, Leipzig, Germany; ^3^Department of Psychology, Università Cattolica Sacro Cuore, Milan, Italy; ^4^Group Cognitive Psychology and Neuroscience, University of Graz, Graz, Austria; ^5^Nuclear Magnetic Resonance Unit, Max Planck Institute for Human Cognitive and Brain Sciences, Leipzig, Germany

**Keywords:** word learning, word representation, depth of encoding, foreign language, gesture, memory, fMRI

## Abstract

Word learning is basic to foreign language acquisition, however time consuming and not always successful. Empirical studies have shown that traditional (visual) word learning can be enhanced by gestures. The gesture benefit has been attributed to depth of encoding. Gestures can lead to depth of encoding because they trigger semantic processing and sensorimotor enrichment of the novel word. However, the neural underpinning of depth of encoding is still unclear. Here, we combined an fMRI and a behavioral study to investigate word encoding online. In the scanner, participants encoded 30 novel words of an artificial language created for experimental purposes and their translation into the subjects’ native language. Participants encoded the words three times: visually, audiovisually, and by additionally observing semantically related gestures performed by an actress. Hemodynamic activity during word encoding revealed the recruitment of cortical areas involved in stimulus processing. In this study, depth of encoding can be spelt out in terms of sensorimotor brain networks that grow larger the more sensory modalities are linked to the novel word. Word retention outside the scanner documented a positive effect of gestures in a free recall test in the short term.

## Introduction

Vocabulary acquisition in a foreign language (L2) is a tedious and time-consuming task that learners usually perform by reading and repeating the words in bilingual lists. Better results in memorization are achieved if words are enriched by pictures (Mayer et al., 2015) and even better by gestures (for reviews, see Macedonia and Von Kriegstein, 2012; Macedonia, 2014). The positive effect of gestures on memory for words and phrases in native language – the *enactment effect* – has been investigated extensively since the 1980s (for reviews, see Nilsson, 2000; Zimmer, 2001) and has been explained by the following different and partially controversial accounts. First, self-performed action leads to the formation of a *motor trace* that drives the enhancement (Engelkamp and Krumnacker, 1980; Engelkamp and Zimmer, 1985; Nilsson et al., 2000; Masumoto et al., 2006; Eschen et al., 2007; Macedonia et al., 2011); second, representing the word by a meaningful gesture produces a kinematic image of the concept that matches an internal representation of the word (Saltz and Donnenwerthnolan, 1981; Kelly et al., 2009; Macedonia et al., 2011); third, multisensory processing during gesture performance increases perception and attention and thereby strengthens memory (Kormi-Nouri, 1995, 2000). Studies attributing the enactment effect to the above reasons additionally address depth of encoding as the factor leading to the memory enhancement (Quinn-Allen, 1995; Tellier, 2008; Kelly et al., 2009; Macedonia et al., 2011; Macedonia and Klimesch, 2014).

Depth of encoding was originally described in Craik and Lockhart’s *levels-of-processing* (LOP) framework (Craik and Lockhart, 1972; Craik and Tulving, 1975). Thereafter memorization consists of three stages: encoding (processing of incoming information), storage (maintenance and representation of the information), and retrieval (recollection of the information for specific purposes) (Atkinson and Shriffin, 1968).

According to the LOP framework, information is processed hierarchically: sensory perception is considered as “shallow” and decays fast. By contrast, semantic processing is considered as “deep”. In deep semantic processing, patterns are recognized, meaning is extracted (Ekuni et al., 2011), and information is kept in memory in a durable way. For example, a word in a foreign language that we only hear is likely to be forgotten because encoded shallowly. In a similar way, vocabulary that we read in lists decays within a short time (Macedonia and Klimesch, 2014). In contrast, encoding becomes deep if vocabulary is learned by selecting it for certain features, i.e., when completing a text or doing other exercises that involve semantic processing (Craik and Tulving, 1975).

The LOP framework describes depth of encoding also in another way. Besides semantic processing, Craik and Tulving (1975) asserted that retention is influenced by the sensorial richness with which verbal material is presented. In other words, adding sensory features to a novel vocabulary item enhances its retention. As documented in the study by Mayer et al. (2015), enriching a novel word in L2 by a picture or by a gesture supports its retention compared to acoustic encoding which is considered as shallow whereby gestures proved to be more efficient than pictures.

Interestingly, also enactment research reconducted depth of encoding through gestures to a multi-component system that drives explicit memory (Engelkamp and Zimmer, 1994). This system consists of a “verbal system”, for input and output, and other “non-verbal”, i.e., sensorimotor systems. When someone encodes a novel word, different systems are engaged. If the novel word is accompanied by an illustration, the visual system, in addition to the verbal one, will process the information (see also Paivio, 2006). Along this line, if a gesture accompanies the word, the motor system will also participate in encoding. Engelkamp and Zimmer (1994) proposed that the different systems create a sensorimotor representation of the word and that this representation is activated during retrieval. In their account, depth of encoding is explained in terms of sensorimotor complexity of information with a particular focus on the motor component present if a new word is encoded through gestures (Engelkamp and Jahn, 2003). According to Engelkamp and Zimmer (1994), the involvement of the motor system would, however, play the major role in memory enhancement.

### Learning Words in a Foreign Language Through Gestures and Depth of Encoding

How are gestures that accompany a word in foreign language related to depth of encoding? How can gestures make verbal information processing deep?

Learners process sensory information on multiple levels if they watch somebody enacting a word or a phrase (Engelkamp and Zimmer, 1994; Klimesch, 1994). Furthermore, if learners perform the gestures themselves, they enhance memory (self-performed task effect, see Engelkamp et al., 1994; Mulligan and Hornstein, 2003). In this case, depth of word encoding is induced first sensorily by enrichment, i.e., by perceiving the gesture and, in a second step, by self-performance.

From a semantic point of view, the match between kinematic image and word semantics leads to deeper encoding than reading or hearing the word. Consider a language teacher illustrating the Japanese word *nomu* (drink) (Kelly et al., 2009) and raising his hand as if holding an invisible cup. Learners observing the teacher will make an involuntary match with an internal template, an image they have stored for the semantics of *drink*. This template can vary in its spatial range to a certain extent, i.e., the gesture can be more or less similar to the internal kinematic image (Kelly et al., 2012).

A match between gesture’s perception and gesture’s representation seems to be present in the learner’s mind. Mismatch paradigms have demonstrated that subjects are susceptible to gestures that do not belong to their inventory and/or do not match their internal kinematic representation of a word (Ozyurek et al., 2007; Willems et al., 2007; Holle et al., 2008; Green et al., 2009). In an fMRI study in foreign language, Macedonia et al. (2011) showed that words that were learned with meaningless, hence not matching, gestures activated a network denoting cognitive control, as in Stroop tasks. This implies that learning a word accompanied by a gesture triggers an internal image, an embodied representation of its semantics.

Hence, we reason that gestures lead to deeper encoding on two paths: first they involve multiple sensory and motor systems in their representation. Second, gestures also induce semantic processing.

### Neural Underpinning for Depth of Encoding

The neural substrate of depth of encoding is not fully understood. The literature in the field mainly considers two possibilities: the “semantic processing” view (Otten et al., 2001; Nyberg, 2002) connects depth of encoding with increased activity in prefrontal and temporal brain areas on the base of the task the subject performs (Nyberg, 2002). An incidental task, for example, the detection of the letter A in a word, recruits sensory (visual) regions. The encoding is shallow and the memory performance is less optimal correspondingly. A task demanding more semantic elaboration, like classifying words as for example living or non-living, engages the prefrontal cortex including the core language regions (BA 10, 45, 46, 47). Similarly, deep elaboration tasks additionally involve left temporal regions (para-hippocampal/fusiform gyri). According to Nyberg (2002), increased activity in these regions is responsible for deeper encoding and leads to better retrievable memory traces.

The other view of depth of encoding attributes the beneficial effects on memory to the recruitment of multiple cortical areas, including motor regions (Klimesch, 1987, 1994; Engelkamp and Zimmer, 1994; Plass et al., 1998; Shams and Seitz, 2008; Shams et al., 2011). In a recent brain imaging study, Macedonia and Mueller (2016) have advanced the hypothesis that depth of encoding through gestures can be connected with the involvement of procedural memory in word learning.

When enriched by gestures, word encoding can potentially exploit both semantic processing and complex sensorimotor perception. These processes need not be mutually exclusive; rather, they could complement each other. Hence, depth of encoding can be an additive process: from shallow sensory perception, to complex sensory perception, and finally to semantic processing.

### The Present Study

Studies conducted to date on the effect of gestures on memory have imaged brain responses after participants had learned the words, i.e., after encoding and maintenance of information in memory in the short and long term (for reviews, see Macedonia and Von Kriegstein, 2012; Macedonia, 2014). Studies so far have investigated retrieval but not encoding itself. These studies have not disentangled the benefit of multisensory encoding from the benefit of sensorimotor training when subjects perform themselves the gestures. In fact, it is conceivable that depth of encoding is given by the sensorimotor repetition itself during learning and not necessarily already in the phase of encoding. On the other side, it is also conceivable that depth of encoding is created in the very first steps of perception. Thus, the way learners encode information can have an impact on performance. If we learn vocabulary by reading, or by watching a trainer performing a gesture that is semantically related to the word, we encode the information, respectively, in a shallow or in a deep way.

In L2 education, gestures accompanying words can be used in a two ways:

(a) In encoding, as a *presentation tool* first performed by the teacher while introducing orally novel texts (Macedonia, 2013);
(b) In training, as a *tool after* presentation; in this phase, learners repeat actively the word and the gestures paired to it.

In the present study, we explore encoding online with gestures as a *presentation tool*. We ask if sensorimotor encoding can enhance behavioral performance compared to reading and reading and hearing a word from the very first moment. The question is relevant because in practice, this knowledge could change the way novel vocabulary is introduced to learners. From a neuroscientific point of view, encoding the word from the very first presentation together with sensorimotor information might lead to the creation of extended networks that store information more efficiently and make it better retrievable. These networks could process the information in a more powerful way than networks consisting of less components (Klimesch, 1994). The outcome of our investigation can be relevant to instruction in order to make vocabulary learning more efficient than it is at present.

The present study wants to shed light on the neural substrate of word encoding with procedures that range from shallow to deep. To our knowledge, our study is the first to measure neural activity during first-stage encoding of words of a foreign language with modalities that grow in complexity and can be connected to different learning procedures in foreign language instruction.

By means of fMRI, we investigate online encoding of novel words through reading (V), audiovisually, i.e., reading and listening to an audio file (AV), and by enriching audiovisual encoding through observed gestures (SMO) with an actress performing a gesture semantically related to the word. These three modalities go from shallow to deep (Klimesch, 1987).

We thus explore the following research questions:

(1) How is stimulus complexity mapped into brain networks?
(2) We hypothesize that complexity of the perceived stimulus is translated into the network extension. As we increment stimulus modalities, functional networks grow larger and from shallow they become deep. In the gesture condition, networks include sensorimotor areas. We thus plan to contrast hemodynamic images of sensorimotor encoding (SM) vs. audiovisual encoding (AV) and audiovisual encoding (AV) vs. reading (V). Also, we will contrast reading (V) with the baseline silence (S).
(3) Is network extension associated with depth of encoding and consequently word retention? We hypothesize that a larger network and particularly a network including sensorimotor areas will impact the depth of encoding. The behavioral outcome of this larger network, compared to less extended networks, will be enhanced word retention.
(4) Do different sensory stimuli involve semantic areas differentially? It is conceivable that with growing depth of encoding, semantic areas are more strongly engaged. In other words, we expect that encoding through observed gestures engages semantic areas more than encoding through reading (baseline) and encoding through reading the word and listening to it.

## Materials and Methods

### Participants

Thirty six right-handed German natives, students of the University of Graz took part in the fMRI experiment. Three subjects had to be excluded for technical reasons, and two failed to complete the experiment. This left 31 participants (thereof 20 females) for analysis with a mean age of 24.35 years, *SD* 3.04. All participants had normal hearing status and normal or corrected vision. They reported no history of neurological or psychiatric illness. They were recruited from the data base of the Psychology Department of the University of Graz. Participants received 10€ compensation for their participation and gave written informed consent prior to the experiment. The study was approved by the Ethics Committee of the University Graz (Austria).

### Stimulus Material

The training materials consisted of 30 three-syllable words in an artificial corpus (Table 1) called Vimmi (Macedonia et al., 2011). Vimmi words were randomly generated by a Perl script according to Italian phonotactic rules. In order to avoid associative learning, we controlled the items for similarity to words in European languages previously learned by the subjects, similarity to proper names of products available on the Austrian and German markets, tautological occurrence of syllables, appearance of strings sounding unusual to German-speaking subjects, and high frequency of particular consonants or vowels. We did this to exclude distinctiveness (Hunt and Worthen, 2006) and bizarreness effects (Baddeley and Andrade, 2000) that might have an impact on word retention. We assigned 30 concrete nouns (everyday objects as *knife, chair, flower*, etc.) and German translations for the Vimmi words arbitrarily. The German nouns were controlled for their frequency of occurrence (familiarity) according to the Vocabulary Portal of the University of Leipzig^1^.

**Table 1:** List of stimuli.

Cond. 1 Visual (V)			
	**Vimmi**	**German**	**English**
1	Nelosi	Reissverschluss	Zip
2	Gelori	Ohrring	Earring
3	Miruwe	Pfeffermühle	Pepper mill
4	Gepesa	Besen	Broom
5	Mebeti	Becher	Cup
6	Atesi	Treppe	Stairs
7	Lofisu	Foen	Hair dryer
8	Serawo	Giesskanne	Watering can
9	Siroba	Seife	Soap
10	Botufe	Taschentuch	Handkerchief
**Cond. 2 Audiovisual (AV)**			
11	Suneri	Geige	Violin
12	Wugezi	Regal	Shelf
13	Mewima	Stempel	Stamp
14	Guriwe	Faden	Thread
15	Sigule	Tempel	Temple
16	Lifawo	Stuhl	Chair
17	Bekoni	Kaffee	Coffee
18	Dafipo	Huegel	Hill
19	Pirumo	Erde	Earth
20	Giketa	Blume	Flower
**Cond. 3: sensorimotor (SM)**			
21	Magosa	Shampoo	Shampoo
22	Uladi	Pullover	Pullover
23	Dirube	Zettel	Sheet of paper
24	Ganuma	Messer	Knife
25	Nabita	Welle	Wave
26	Mesako	Telefon	Telephone
27	Midaro	Spiegel	Mirror
28	Raone	Fernbedienung	Remote control
29	Motila	Banane	Banana
30	Nukile	Poster	Poster

*Words used during encoding in the scanner, their translation into German for the participants, and into English for the readers.*

**FIGURE 1:**
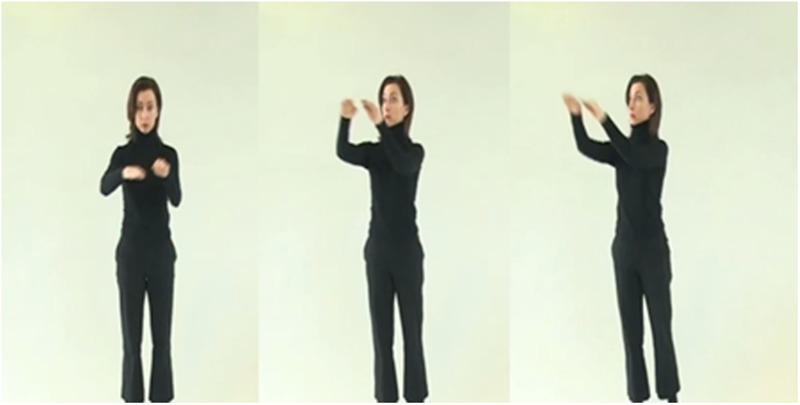
Screenshots of a video with the actress while performing the gesture for the word “stair”.

The 30 Vimmi words were spoken and recorded by a female German speaker, with each audio file having a length of approximately 1 s. We also recorded 10 videos clips for the sensorimotor (SMO) condition of an actress performing a gesture associated with the German translation of each of 10 Vimmi words (see an example in Figure 1), with an average duration of 4.7 s.

### fMRI Experiment: Word Encoding in the Scanner

Stimulus presentation and scanner triggering were controlled by a computer outside the scanner room using the software Presentation (version 16.0, Neurobehavioral Systems^2^). Participants lay supine in the scanner. The written Vimmi words, their translation in German, and the videos were presented via an LCD projector onto a back-projection screen mounted at the participant’s feet. Participants viewed the contents of the screen over a mirror mounted on top of the head coil. The Vimmi audio files were presented using an in-ear headphone system (Earplug, NordicNeuroLab AS, Norway).

#### Procedure

The training materials (30 Vimmi words and their translations as shown in Table 1) were presented to the subjects under three conditions:

(1) Visual (V) condition. Written word in Vimmi with German translation (items 1–10);
(2) Audiovisual (AV) condition. Written word in Vimmi with German translation and acoustic presentation of the Vimmi word (items 11–20);
(3) Sensorimotor observation (SMO) condition. Written word in Vimmi with German translation, acoustic presentation of the Vimmi word, and video with an actress performing an iconic gesture semantically related to the word (items 21–30).

Subjects were instructed to memorize the Vimmi words together with their German translations. Subjects were informed that they would perform retention tests outside the scanner after the encoding phase.

The experiment began in the scanner with the instructions shown for 30 s. After a 10-s black screen, the first block started. The blocks for the different conditions were presented in a randomized order. In each trial, a written word in Vimmi appeared at the lower part of the screen with its German translation underneath. It remained visible for 7 s, i.e., V condition. In the AV condition, an audio file played with the onset of the written stimulus. In the SMO condition, a video of the gesture augmented the written and audio stimuli. Each item was presented for 7 s, with an inter-stimulus interval of 10 ± 4 s that varied randomly in 500 ms steps. Due to the repetition time of 2130 ms (for details, see the following chapter), three functional volumes were completely acquired during stimulus presentation. During the inter-stimulus interval (i.e., in the time between the presentation of the stimuli), a blank screen appeared.

**FIGURE 2:**
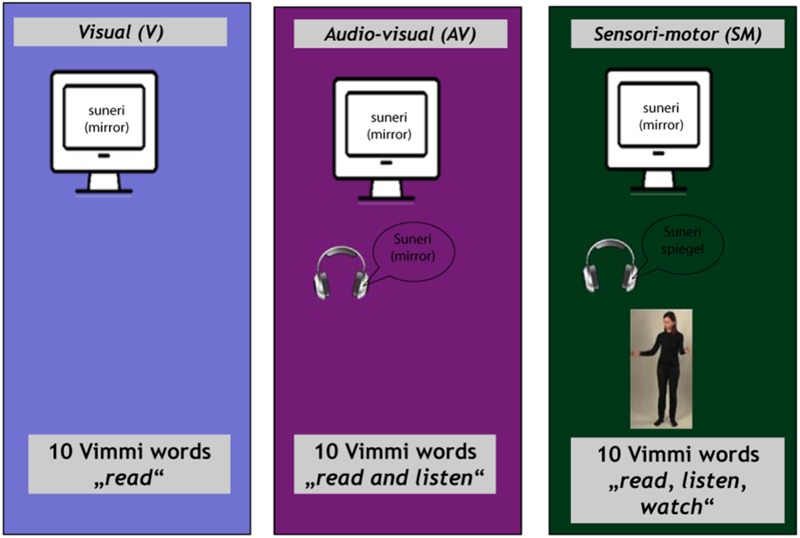
Training conditions in the scanner with instructions.

The items were divided into blocks of 10 items, presented separately for each of the three learning conditions. Each block was shown three times, with the 10 items for each condition randomized within the block, for a total of nine blocks and 90 trials. Altogether, every vocabulary item was presented three times in its respective learning condition. The entire encoding part of the study lasted approximately 25 min (Figure 2).

#### MRI Data Acquisition

Imaging was performed using a 3T whole-body system Siemens Trio scanner with an echo-planar capable gradient system and a Siemens-issued 32-element coil. For the functional experiment, we used a T2^∗^-weighted echo-planar imaging (EPI) sequence (flip angle 90°, repetition time 2130 ms, echo time 31 ms, image matrix = 64 × 64, 32 axial slices, in-plane resolution: 3 mm × 3 mm, slice thickness: 3 mm, 1 mm gap), which is sensitive to the brain oxygen level dependent (BOLD) contrast. Due to the variable inter-stimulus interval as described above, the total length of the functional experiment varied slightly between the subjects. On average, 746 functional volumes were used resulting in a total experiment length of 26.5 min. The heads of the participants were stabilized with foam padding. Participants were protected from the scanner noise by earplugs embedded in the in-ear headphone system.

For image registration and normalization, a T1-weighted high-resolution image was acquired prior to the functional images. For each subject, one volume was obtained using a three-dimensional magnetization-prepared rapid gradient-echo (MPRAGE) sequence. Acquisition parameters were chosen using a flip angle of 9°, an echo time of 2.07 ms, an inversion time of 900 ms, a repetition time of 1560 ms, and a bandwidth of 230 Hz/Px. A sagittal slice orientation was chosen with 176 slices and an in-plane field-of-view with 256 mm × 256 mm. The nominal image resolution was set to 1 mm × mm 1 × 1 mm with a final image matrix of 176 × 256 × 256.

#### MRI Data Analysis

Data analysis and pre-processing were performed with SPM8 (Wellcome Department of Cognitive Neurology, London, United Kingdom). Standard processing included realignment, slice-time correction, and normalization to the Montreal Neurological Institute (MNI) space based on the unified segmentation approach (Ashburner and Friston, 2005). After normalization, the resulting voxel size of the functional images was interpolated to an isotropic voxel size of 3 mm × 3 mm × 3 mm. Images were finally smoothed with a Gaussian kernel of 8 mm FWHM. Statistical analysis was performed on the basis of the general linear model as implemented in SPM8. The motion parameters derived from the realignment procedure were entered into the model as parameters of no interest. A high-pass filter (cut-off frequency 1/120 Hz) was used to remove low frequency drifts. No global normalization was used.

A model with three conditions (visual, audiovisual, and sensorimotor) was used with SPM8 and an event-related design. Here, the delta function of the event onsets (corresponding to the onset of each stimulus presentation) for each condition was convolved with the canonical form of the hemodynamic response and its first temporal derivative. Finally, on a single-subject level, contrast images were generated by computing difference images between parameter estimates. Three types of contrast images were obtained (i) subtracting the visual condition (V) from the implicit baseline silence (S), (ii) subtracting the visual condition (V) from the audiovisual condition (AV), and (iii) subtracting the audiovisual (AV) with the sensorimotor condition (SMO). The contrast images calculated for individual subjects were entered into second level random effects analyses (Friston et al., 1999). Finally, in order to correct for multiple comparisons, a family-wise error (FWE) correction was applied on resulting statistical parametric maps with *p* < 0.05 on the cluster level. Here we used a threshold of a minimum cluster size of 30 voxels. The FWE correction was performed using SPM8 based on the Gaussian random field theory.

#### Results

First, we extracted the implicit baseline silence (S) from the visual condition. During the task, participants showed an extended network mapping the reading (written word) and relating it to the word in the native language. It includes the inferior frontal gyrus, the SMA, and the vermis in the left hemisphere. Bilaterally, we found the inferior frontal gyri, the fusiform gyri, the insulae, and the right hippocampus. The right superior temporal pole, the anterior and the posterior cingulate gyri, as well as the superior frontal gyrus participate in the network.

Second, we subtracted the visual (V) from the audiovisual condition (AV). This contrast shows activity in the temporal lobes bilaterally, i.e., right superior temporal gyrus, left middle temporal gyrus, the right inferior frontal gyrus, as well as in the left hippocampus.

Third, we contrasted the audiovisual (AV) with the sensorimotor condition (SMO). Bilaterally, the motor cortices and the inferior parietal lobules were active during encoding. The right superior temporal gyrus, the right vermis, as well as the left fusiform gyrus completed the network.

**FIGURE 3:**
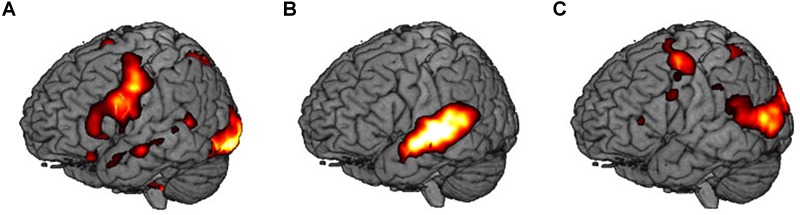
Main contrasts in brain activity: **(A)** visual encoding, V vs. S, **(B)** audiovisual encoding, AV vs. V, and **(C)** sensorimotor encoding, SMO vs. AV.

Areas that were significantly activated during encoding are shown in Table 2 and Figure 3.

**Table 2:** Brain areas activated in the different encoding conditions.

Hemisphere		*x*	*y*	*z*	*k*	*Z*
	**Contrast: baseline – visual (V) vs. silence (S)**					
Right	Fusiform gyrus	38	-74	-14	2756	Inf.
Left	Fusiform gyrus	-38	-66	-16	2544	Inf.
Left	Inferior frontal gyrus	-48	14	26	1760	7.55
Left	Cerebellum, vermis	0	-54	-40	141	6.90
Left	SMA	-2	8	56	1253	6.77
Left	Insula	-32	22	-4	830	6.72
Right	Insula	32	28	-2	728	6.60
Right	Hippocampus	22	-30	-2	1549	6.51
Right	Inferior frontal gyrus	46	10	28	932	6.43
Left	Superior parietal lobule	-28	-60	52	739	6.42
Right	Superior parietal lobule	28	-62	52	733	6.25
Right	Superior temporal pole	46	12	-22	123	6.01
Right	Anterior cingulate gyrus	4	-32	34	163	5.70
Left	Middle temporal gyrus	-58	-44	10	89	5.69
Right	Middle temporal gyrus	58	-2	-16	48	5.65
Right	Posterior cingulate gyrus	8	-2	32	46	5.51
Right	Superior frontal gyrus	32	0	66	432	3.93
	**Contrast: audiovisual (AV) vs. visual (V)**					
Right	Superior temporal gyrus	64	-28	4	3902	Inf.
Left	Middle temporal gyrus	-64	-14	-2	3791	Inf.
Left	Hippocampus	-12	-26	-8	45	5.86
Right	Inferior frontal gyrus	38	16	26	345	4.94
	**Contrast: sensorimotor observation (SMO) vs. audiovisual (AV)**					
Left	Fusiform gyrus	-22	-82	-10	6389	Inf.
Right	Superior temporal gyrus	58	-40	16	1509	7.07
Left	Precentral gyrus	-36	-4	52	388	6.83
Right	Precentral gyrus	46	2	50	374	6.35
Left	Inferior parietal lobule	-22	-52	54	299	6.25
Right	Inferior parietal lobule	30	-40	52	409	5.60
Right	Cerebellum: vermis	0	-30	-4	56	5.47

*Statistical parameter maps were thresholded at *p* < 0.05 FWE-corrected. Coordinates are reported as given by SPM8 (MNI space). *k* = cluster size, *Z* = *Z* value for the maximally activated voxel of the cluster.*

#### Discussion

The results of the fMRI contrast analyses show word processing on the different levels of depth while participants encoded the novel words visually, audiovisually, and by observing the gestures related to the words.

In the first contrast S vs. V, we expected an extended network that maps visual word encoding as well as regions involved in processing the visual written input. We found both, the extended network, and bilaterally the fusiform gyri active in reading tasks and in letter processing (McCandliss et al., 2003). The hippocampus besides memory tasks subserves also functions such as binding and multimodal integration (Breitenstein et al., 2005; Straube et al., 2008). This could be here the case. At a closer look, the activity map reveals the involvement of ventral occipital regions surrounding the fusiform gyrus. This might support the view that reading is accomplished in a ventral occipital temporal network of visual areas (not only focally in the fusiform gyrus), as indicated in a review by Wandell (2011), and more recently by Hannagan et al. (2015). The role of the superior frontal gyrus, here the lower portion of Brodmann area 6, adjacent to primary motor cortices involved in speech articulation, could be that of simulating sound production while participants read the words (Dietz et al., 2005). The left inferior frontal gyrus, fundamental to language tasks (Friederici, 2011; Krönke et al., 2013) in its ventral portion, and to word encoding (Wagner et al., 1998; Kirchhoff et al., 2000) has been claimed to mediate also semantic retrieval (Abutalebi, 2008), and semantic integration between language and gesture (Ozyurek, 2014). This might be the case for our data. In fact, all these areas converge into a universal reading network that orchestrates and reflects the different components of the task (Price, 2012; Rueckl et al., 2015).

During reading, participants learned novel words in Vimmi and connected them with words in their native language. The left middle temporal gyrus, an area engaged in fast mapping of new words (Rodriguez-Fornells et al., 2009), semantic processing (Levy et al., 2004), conceptual semantic representation (Binder and Desai, 2011), and declarative memory (Squire et al., 2004), was also active in the network, together with the anterior and posterior cingulate cortex. The anterior cingulate gyrus, one of the core regions mediating cognitive control, is considered critical to novel word acquisition (Abutalebi and Green, 2007; Raboyeau et al., 2010). As described in a longitudinal fMRI study on L2 word processing (Grant et al., 2015), the anterior cingulate cortex (ACC) interacts with the middle temporal gyrus, a region also present in our data. The ACC detects unfamiliar patterns in the letter sequence and monitors language conflict between native and foreign language (Abutalebi et al., 2012). In a study by Grant et al. (2015), participants had to judge on first and second language words, as well as interlingual homographs, while lying in the scanner, a task very similar to the one in our study. In the Grant study, the results of region of interest and connectivity analyses showed that regular learning of L2 words changed activation patterns between the ACC and the middle temporal gyrus. While activation decreased in the ACC, it increased in the middle temporal gyrus. This indicates that the more familiar the words become, the less the ACC responds. Similarly, the more the words find access to the middle temporal gyrus, the more the latter will react upon word recognition. On this base, our data could also mirror the interaction between ACC and middle temporal gyrus at the very beginning of learning, when subjects were confronted for the first three times with the foreign language words and their translations. The posterior cingulate gyrus (BA 31) might have responded to visual stimuli during reading (Vogt et al., 1992) but also have been involved in processes of episodic memory (Rolls, 2015). In this contrast, we also find activity in the insula, recently detected in word processing (Zaccarella and Friederici, 2015) and engaged in manifold language tasks (for a review, see Oh et al., 2014).

In the contrast AV vs. V, we found the “classical” network for language processing with the participation of the superior and middle temporal gyri essential in auditory word processing (Davis et al., 2009; Segawa et al., 2015), and the right inferior frontal gyrus. In a meta-analysis of neuroimaging studies on the functions of the inferior frontal gyrus, Liakakis et al. (2011) relate this area to fine movement control and working memory. In our study, the right inferior frontal gyrus could be connected to unintentional subvocalization and to memorizing the novel words, as indicated in a similar study by Krönke et al. (2013). Interestingly, in the audiovisual condition, the left hippocampus was significantly more involved than on reading only. This structure has been identified as particularly relevant in word learning, especially during the initial encoding of words (McClelland et al., 1995; Davis and Gaskell, 2009).

In the SMO vs. AV contrast, considering that subjects watched videos with an actress performing gestures, we expected our data to be in line with other studies targeting premotor cortices during movement observation (Buccino et al., 2001; Buccino et al., 2004a,b; Straube et al., 2008; Ogawa and Inui, 2011) and connecting them to the mirror neuron system implicated in action recognition (Rizzolatti, 2015). Despite this, we found bilateral activity in the primary motor cortices and in the parietal lobules. Recently, fMRI studies reported these brain areas also during action observation (Aridan and Mukamel, 2016; Gatti et al., 2016) as belonging to a mirror neuron network active in motor learning tasks that include observation as well as imitation and execution (Sale and Franceschini, 2012). Activations located in the parietal lobules are possibly due to the actress’ body observation (Hodzic et al., 2009). In fact, participants processed the actress’ image while she performed the gestures. Along this line, activity located in the inferior parietal lobules is possibly due to the actress’ body observation (Hodzic et al., 2009). Participant processed in fact her image while she performed the gestures. Furthermore, we found additionally activity in the cerebellar vermis: this brain region might subserve here visual motion processing (Cattaneo et al., 2014).

In the fMRI contrast analyses, we examined the neural activity involved in encoding novel words in a foreign language according to three conditions with growing complexity. The analyses show that the network processing the words also grows in complexity. The network mirrors the modalities added progressively: a basic network engaged in encoding through reading in the visual condition is enlarged by auditory cortices in the audiovisual condition, by motor cortices, and the parietal lobules in the condition with gesture observation. Taken together, the network that encodes novel verbal information by observing gestures seems to connect a set of regions in the left hemisphere: canonical language areas in inferior frontal regions, (pre)motor cortices, and the hippocampi.

Our results are in line with neuroimaging studies that have investigated single word learning (Paulesu et al., 2009; Ye et al., 2011). Despite the different paradigms, these studies have found the recruitment of brain tissue in the left hemisphere, more specifically in the inferior frontal gyrus, the parahippocampal region, and the fusiform gyrus. A study by Straube et al. (2008) explored the retention of Russian sentences accompanied by unrelated or metaphoric gestures by means of a recognition task. Encoding of verbal information with metaphoric gestures, a task similar to the one that our subjects had to accomplish, was associated with neural activity in the left inferior frontal gyrus, the premotor cortex, and the middle temporal gyrus. The hippocampus contributed to retention and correlated with performance. The fMRI study by Straube et al. (2008) yields comparable results for the encoding condition with metaphoric gestures in brain activity with our study for the condition SMO.

### Behavioral Experiment: Word Retrieval Outside the Scanner

#### Procedure

After the fMRI session, participants spent a 5-min break in a room adjacent to the scanner. In this room, subjects completed four different pencil-to-paper tests, as described in the next sections.

#### Free Recall Tests for German and Vimmi

We gave participants an empty sheet for each language (German and Vimmi) and instructed them to write as many items as possible in each of the two languages (only German or only Vimmi). Participants had 5 min for each of the free recall tests, for a total of 10 min.

#### Paired Free Recall Test

Participants were given an empty sheet and instructed to write as many items as possible in both languages (pairs). In this test, items had to be matched (i.e., German and the corresponding word in Vimmi or vice versa). The paired free recall test lasted 5 min.

#### Cued Recall Tests

We gave participants a randomized list of the 30 trained items to be translated from German into Vimmi (duration 5 min) and then another randomized list of the same words to be translated from Vimmi into German (duration 5 min). The order of the translation from one into the other language alternated from participant to participant.

#### Follow-Up Tests

We also sought to know how encoding influences decay in time. Therefore, after approximately 45 days, we asked subjects to participate in a follow-up test. We emailed our participants a link to anonymous word retrieval tests that would document their memory performance. Out of 31 participants, 18 accepted the invitation. The online tests were realized with the Qualtrics software, version 56531, in the Qualtrics Research Suite^3^. Participants completed online the same tests that were administered after encoding, i.e., free recall (German, Vimmi, and paired) and cued recall tests in both translation directions. Each test was time-locked: after a time limit of 5 min, the current test disappeared and the next test was presented. After completion, results contained in the log files were entered into SPSS for behavioral statistical analysis.

#### Statistical Analyses

**FIGURE 4:**
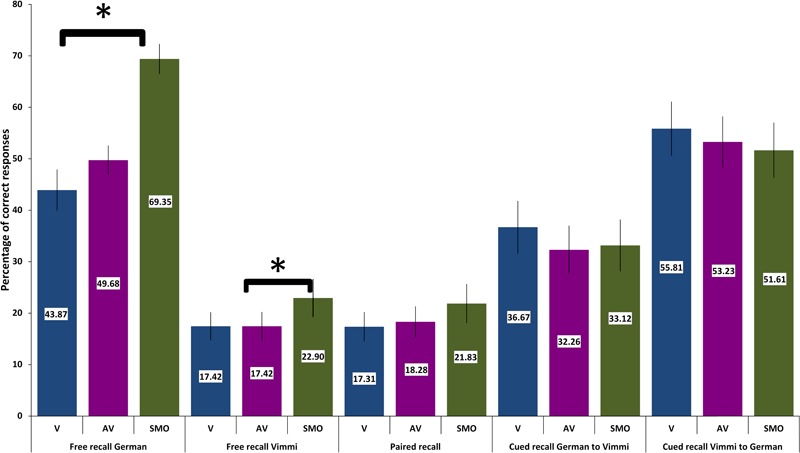
Memory performance in the pencil-to-paper tests immediately after encoding. Error bars indicate one standard deviation. ^∗^*p* < 0.05.

For each memory task and for each experimental condition, a performance index was calculated as the percentage of correctly recalled items over the total number of items. Then the performance scores were entered in a repeated measures ANOVA, with the conditions visual (V), audiovisual (AV), and sensorimotor (SMO), as within-subjects factors. *Post hoc* contrasts (within subjects simple contrasts) between conditions were computed when needed. Descriptive statistics results are reported in Figure 4, 5 (immediate recall and follow-up performances, respectively).

**FIGURE 5:**
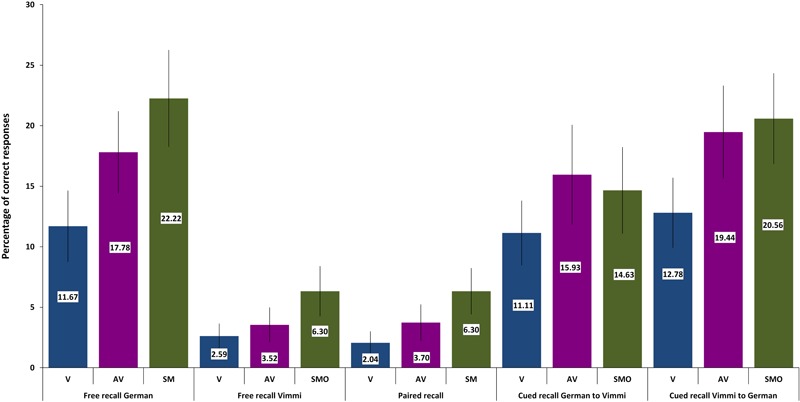
Memory performance in the pencil-to-paper test after ca. 45 days. Error bars indicate one standard deviation.

Finally, in order to assess the influence of the different conditions on neural mechanisms related to depth of encoding, we conducted correlation analyses between brain and behavioral data. We ran two parametric analyses based on the contrast SMO vs. AV. As a regressor for the second level analysis, we entered the behavioral test results showing significant differences among encoding conditions, i.e., (1) the results from the free recall test in German and (2) the results from the free recall test in Vimmi (Table 3). The threshold for these two analyses was set to *p* < 0.005 uncorrected, reporting only clusters with a minimal cluster size of 30.

**Table 3:** Behavioral average performances in free Vimmi and free German.

Subject	Free German	Free Vimmi
enc_01	63,33	28,89
enc_02	56,67	4,44
enc_03	43,33	18,89
enc_04	80,00	36,67
enc_05	60,00	44,44
enc_07	66,67	25,56
enc_08	53,33	2,22
enc_09	80,00	42,22
enc_10	63,33	3,33
enc_11	50,00	18,89
enc_12	70,00	23,33
enc_15	36,67	13,33
enc_16	46,67	0,00
enc_18	40,00	6,67
enc_19	60,00	20,00
enc_20	23,33	5,56
enc_21	46,67	12,22
enc_22	50,00	16,67
enc_24	63,33	18,89
enc_25	63,33	20,00
enc_26	60,00	11,11
enc_27	50,00	18,89
enc_28	70,00	54,44
enc_29	40,00	5,56
enc_30	60,00	21,11
enc_31	40,00	8,89
enc_32	20,00	0,00
enc_33	56,67	31,11
enc_34	56,67	7,78
enc_35	63,33	54,44
enc_36	50,00	21,11

#### Results

##### Memory performance

Figure 4 shows that participants recalled more words in German than in Vimmi. For the *free recall* test in *German*, data underlined that performance differed significantly among conditions [*F*(2,60) = 28,26, *p* < 0.001, η^2^ = 0.48]. Sensorimotor encoding proved to be significantly superior to both visual [*F*(1,30) = 42.03, *p* < 0.001; η^2^ = 0.59] and audiovisual encoding [*F*(1,30) = 37.92, *p* < 0.001; η^2^ = 0.56]. In the free recall test in Vimmi, memory performance again differed depending on the type of encoding [*F*(2,60) = 3.38, *p =* 0.04, η^2^ = 0.1], with sensorimotor encoding proving superior to visual [*F*(1,30) = 5.23, *p* = 0.03; η^2^ = 0.15] but not to audiovisual encoding [*F*(1,30) = 3.93, *p* = 0.06].

In the *paired free recall test*, performance did not differ among conditions [*F*(2,60) = 1.94, *p* = 0.15]. In the *cued recall tests from German into Vimmi* and *Vimmi to German*, memory results did not differ with regard to the condition under which subjects had learned [German to Vimmi: *F*(2,60) = 0.78, *p* = 0.46; Vimmi to German: *F*(2,60) = 0.76, *p* = 0.47]. In the follow-up tests, the mean values by condition (Figure 5) indicate that overall the recall rates were very low (ranging from about 2 to 22%). Nevertheless, having a look at the descriptive data, interesting differences emerge if we compare the encoding conditions. In *German and Vimmi free recall* and in *paired free recall*, participants scored worst for words encoded by mere reading (V). For words encoded audiovisually (AV), they did better, and best memory results were obtained through sensorimotor encoding. In particular, the difference between the V and SMO conditions in the *German and Vimmi free recall* tests is worth noting. Participants could retrieve nearly twice as many words encoded with gestures than words learned by mere reading. In the *paired recall test*, participants retrieved three times as many words learned in the SMO than words learned in the V condition. However, due to the high variance in performance among subjects, only the *German free recall* test nearly reached significance [*F*(2,34) = 3.19, *p =* 0.054, η^2^ = 0.19]. In both the cued recall tests, memory performance did not differ among encoding conditions, i.e., from *German into Vimmi* [*F*(2,34) = 2.09, *p* = 0.14] and *from Vimmi into German* [*F*(2,34) = 2.64, *p* = 0.09].

#### Correlations Between Brain Activity and Behavior

The parametric analysis relating the contrast SMO vs. AV with the mean performance of each single subject in the German free recall and Vimmi free recall tests gave further insights in the neural mechanism of depth of encoding. We hypothesized that the results would parametrically mirror the brain patterns of the contrast, hence possibly involve the motor cortices and/or the parietal lobules or parietal regions involved in semantic encoding.

The correlation with the performance score from the *free recall test in German*, the subjects’ native language, shows activity in a number of regions with the biggest area located in the left inferior temporal gyrus. This area is considered to be involved in a large cortical network performing semantic storage (Whitney et al., 2010). During word encoding in an event-related and in a blocked fMRI paradigm, Wagner et al. (1998) measured brain activity. They found that the capacity to memorize verbal information is related to the magnitude of activation in canonical language areas (left prefrontal cortices) as well as in left temporal regions including the inferior temporal gyrus. Similar results were achieved in a study by Kirchhoff et al. (2000) during a semantic task.

The correlation with the performance data from *the free recall test in Vimmi*, the foreign language to the subjects, yielded different results. We found activity in the extra-striate cortex. Also larger areas of activity were present in the left hippocampus and in the left parahippocampal gyrus. These regions are critically involved in memorization tasks and are part of the network for word encoding found in the study by Wagner et al. (1998). We can only speculate on the reason why activity in these areas correlates more with Vimmi word encoding than with German word encoding. Considering that new words in the foreign language need to be stored as sequences of phonemes and graphemes, the hippocampus and the parahippocampal gyrus might perform with larger amplitude because they are more engaged in the Vimmi than in the German task.

Altogether, the correlations between brain activity and behavioral results only partially meet our hypotheses. Expecting a recruitment of semantic areas, we found parametric activity in the left inferior temporal gyrus only for the German words. The Vimmi words recruited canonical memory structures, possibly because of the stronger memory effort needed in order to store grapheme and phoneme sequences in the foreign language.

#### Discussion

In this combined fMRI and behavioral study, participants encoded 30 words of Vimmi in the scanner according to three conditions (V, AV, and SMO) with 10 words for each condition. The encoding was brief, with three repetitions per word.

We expected that enriching a word with multiple and sensorimotor information behaviorally would enhance its memorability in the short term and in the long term. We obtained significant results in the free recall test in German and Vimmi immediately after encoding. After approximately 45 days, only the free recall test in German nearly reached significance.

In sum, encoding with sensorimotor input led to superior results in the free recall tests in the short term.

It is conceivable that under “normal” conditions, i.e., if learning had occurred outside the scanner, behavioral performance might have matched other studies showing that enriched encoding supports memory for words in L2 significantly. In fact, it is likely that learning in the scanner influences performance. Lying supine inside a noisy “tube” is a novel, uncomfortable, and distracting situation for participants. According to Peelle (2014), noise from the scanner reduces sensitivity to acoustic experimental stimuli, requiring additional resources in executive functions while processing. More significantly, scanner noise can alter both auditory and non-auditory brain networks hence compromise the expected results. In our study, we made use of in-ear headphones. According to the producer, they provide “sufficient” noise insulation and ensure good quality for the presentation of auditory stimuli. The influence of the scanner noise is equally present in all learning conditions (V, AV, and SMO). All conditions are thus potentially equally affected by the scanner noise. We chose a standard EPI sequence with a moderate noise level and not continuous EPI sequences with reduced acoustic noise. An alternative to our analyses could have been a finite impulse response model as described in Peelle (2014).

Although subjects were exposed to only three repetitions, in order to separate encoding from storage, from a LOP perspective, retention was high if compared to similar studies. In fact, Macedonia and Knösche (2011) trained participants on a similar experimental design for 4 days, 3 h per day. Results became significant only on days 3 and 4, after a very high number of repetitions. Interestingly, also in that study, subjects performed best in German free recall at all time points. This might be the case because German free recall is the easiest task to accomplish. Once a concept’s semantics is clear, learners already have the “label” for the concept (the word). Learners need not memorize novel sound sequences that do not match syllable structure and sounds of the native language. In that study, after 45 days, in all free recall tests, words encoded with observed gestures scored better than in the other conditions and memory results did not mirror those produced immediately after encoding. In other words, even if in the present study the exposure to stimuli was passive and for a very low number of repetitions, results confirm that sensorimotor encoding is crucial for memory processes.

In our study, contrarily to the majority of the studies in the field, subjects only observed and did not enact the items to be learned. Since the beginning of enactment research, it has been known that self-performing the gestures leads to better memory results than only observing them. This was documented in a seminal study by Cohen (1981), in which the author called the effect unambiguously *subject-performed task effect*. At that time, this issue applied to memory for words in L1. In L2 learning, Macedonia and Klimesch (2014) compared the retrieval of 18 words encoded audiovisually and retrieval of 18 words learned with gestures over 14 months. Although the number of repetitions was also very low (eight repetitions on day 1 and four repetitions on day 8, altogether 12 repetitions) memory performance was highly significant for words encoded through gestures at all times. Particularly impressive were the results after 14 months. Whereas participants had nearly forgotten all words encoded audiovisually (mean retrieval performance 1.15%) participants still could recall 10.45% of the words encoded with self-performed gestures. Although speculative, we reason that best memory results can be achieved if learners encode by watching the gestures and thereafter perform the gestures by themselves. To shed more light on this aspect, further studies should be conducted in order to confirm the possible connection between encoding and active processing.

#### Correlations Between Brain Activity and Behavior

We considered the SMO condition to allow more depth of encoding. The contrast analysis SMO vs. AV showed that encoding with gestures elicited greater signal intensity in the motor cortices bilaterally and in the parietal lobules. In this line, for better memory performance, we expected more engagement of the motor system. Instead, the correlation analyses indicated greater activity in language and memory areas. Similarly, in a study by Macedonia et al. (2010), higher memory performance by means of gestures was hypothesized to be related to engagement of the motor system. However, correlation analyses between significant brain activity and behavioral performance revealed in that study activity in the left angular gyrus (BA 39), and in the right extra-striate-cortex (BA 19). The left angular gyrus is described in the literature as a brain area subserving integrative functions in word processing as well as semantic integration (Binder et al., 2009). In the present study, the portion of the extra-striate-cortex revealed by the correlation analyses is considered in its functionality as a posterior extension of the angular gyrus. So, both studies seem to have a connection which should be elucidated with further experiments. At present, we only see the possibility of a stronger functional link between sensorimotor encoding and memory areas in the brain which seems to be stronger than for visual or audiovisual encoding.

## Conclusion

For the first question posed in the introduction, i.e., on how stimulus complexity is mapped into neural networks, our data show that networks grow incrementally in complexity under the three encoding conditions. The growth reflects the sensorial stimuli processed during encoding. For the SMO condition, the one that we considered to be the most efficient, the network includes bilaterally the primary motor cortices, the cerebellum, and the inferior parietal lobules. The participation of the motor system could indicate that procedural memory is engaged in encoding even if participants only observed the stimuli and did not perform the gesture. This could explain why gestures – even if observed during encoding lead to better memory performance.

The second question, regarding network extension connected to depth of encoding and impact on retention as suggested in Craik and Tulving (1975) and Engelkamp and Zimmer (1994), is partly supported by our behavioral data. The SMO condition is associated with significantly better retention only in the free recall tests in both German and Vimmi in the short term.

Our imaging data answer the third question about the participation of semantic cortices in encoding: the network described in the first contrast silence vs. visual encoding includes the middle temporal gyrus, an area involved in deep semantic processing. However, the literature does not describe the V condition leading to particular semantic elaboration of the word. Furthermore, our data show that reading a word does not have a positive impact on retention as AV and SMO encoding. Here, engagement of the temporal areas might reflect the bilingual presentation in the scanner, and possibly the semantic processing of words in the native language and/or connecting words in L2 to words in L1 (Grant et al., 2015). This is to say that the V condition activated semantic cortices and not the SMO condition as expected. It is contradictive to our expectations and it does not provide evidence that gestures if only observed can engage brain tissues engaged in semantic processing. On the other side, it must be considered that it could be very likely that we do not see the activity in semantic cortices for SMO because the SMO was isolated by subtracting the AV from it. In other words, activity in semantic cortices could have been removed by the fMRI analysis. This is one limitation in the subtraction method: information on shared processing goes lost.

The correlation analysis between the brain data related to the SMO condition and the behavioral free recall tests showed the involvement of the left inferior temporal gyrus, a semantic area active in word encoding, for German words. Vimmi words engaged parametrically more the hippocampus and the parahippocampal gyrus.

In sum, our study suggests that observation of a gesture connected to a word in L2 can make its encoding deeper than only reading the word or reading and listening to it. This seems to be due to the engagement of complex sensorimotor networks more than to deep processing in semantic areas. Complex sensorimotor encoding is associated with better memory results than procedures involving fewer senses and not engaging the motor system.

Hence, although traditional bilingual education mainly employs listening and comprehension activities during encoding of novel verbal material, it could be fruitful to introduce sensorimotor enrichment in L2 word learning and the use of gestures in bilingual education. Further studies in the field should disentangle the impact of gestures in L2 practice with regard to the different phases of learning: from encoding to training with or without consolidation phases in order to make L2 word acquisition more efficient and retention durable.

## Author Contributions

MM conceived the project and contributed to experimental design, stimulus material, behavioral and fMRI data collection, and paper writing. MM and CR organized the project. CR contributed to behavioral and fMRI data collection, behavioral data analysis, paper and writing in behavioral section. AI contributed to fMRI data analysis. KM contributed to critical discussion of data and manuscript. CR, MM, and AI created tables and figures and contributed to review and critique.

## Conflict of Interest Statement

The authors declare that the research was conducted in the absence of any commercial or financial relationships that could be construed as a potential conflict of interest. The reviewer JC and handling Editor declared their shared affiliation.

## Footnotes

1 http://wortschatz.uni-leipzig.de/

2 http://www.neurobs.com

3 http://www.qualtrics.com
